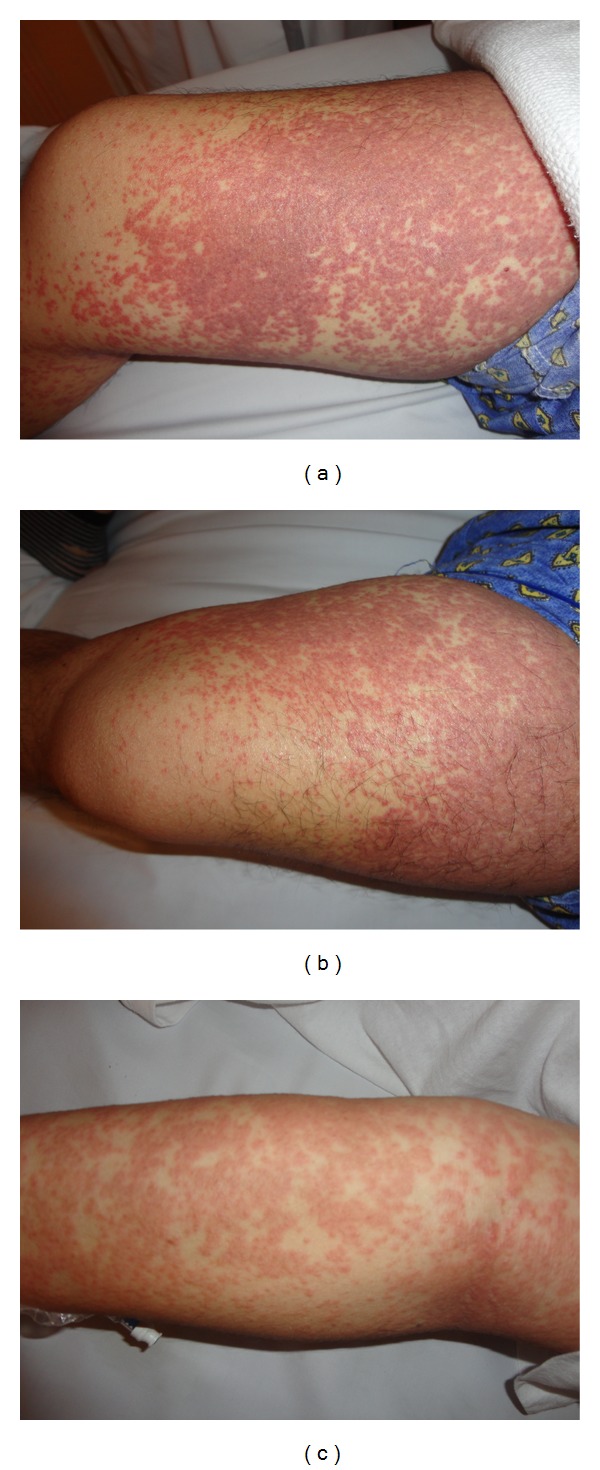# Toxic Epidermal Necrolysis Associated with Antiepileptic Drugs and Cranial Radiation Therapy

**DOI:** 10.1155/2013/415031

**Published:** 2013-07-30

**Authors:** Shereen Elazzazy, Taghrid Abu Hassan, Ashraf El Seid, Cicy Mary Jacob

**Affiliations:** ^1^Pharmacy Department, National Center for Cancer Care and Research NCCCR, Hamad Medical Corporation, Doha, Qatar; ^2^Oncology Hematology Department, National Center for Cancer Care and Research NCCCR, Hamad Medical Corporation, Doha, Qatar

## Abstract

Case reports on the development of toxic epidermal necrolysis (TEN) associated with concurrent administration of phenytoin with cranial radiation therapy (Ahmed (2004), Criton et al. (1997), and Rzany et al. (1996)), but reports about erythema multiforme, which can develop in patients treated with levetiracetam and cranial irradiation, are very limited. This paper presents evidence that TEN may be induced by concurrent use of radiation with both phenytoin and levetiracetam. Our case is a 42-year-old male patient, a case of gliosarcoma who developed purpuric dermatitis associated with phenytoin when combined with cranial radiation therapy; although phenytoin was discontinued and switched to levetiracetam, the patient had more severe symptoms of toxic epidermal necrolysis (TEN) on levetiracetam; the patient improved with aggressive symptom management, discontinuation of antiepileptic drugs (AEDs), and holding radiotherapy. Although TEN is a rare toxicity, physicians should pay a special attention to the monitoring of brain tumor patients on antiepileptic prophylaxis during cranial irradiation; furthermore, patients should be counselled to notify their physicians if they develop any new or unusual symptoms.

## 1. Background

Toxic epidermal necrolysis (TEN, Lyell disease) is a potentially life-threatening condition associated with considerable morbidity and mortality; it is an exfoliative disease and results in full-thickness damage to the epidermis, characterized by a widespread bullae formation with epidermal necrosis and idiosyncratic of the skin and mucous membranes. TEN mainly occurs in adults and is often attributable to drug sensitivity and considered to be a severe form of Stevens-Johnson syndrome (SJS) or erythema multiforme [1]. TEN is linked to drug exposure in up to 90% of the cases [1, 2]; anticonvulsants, antibiotics, allopurinol, and nonsteroidal anti-inflammatory drugs may cause this disease [2–5]. Radiation therapy may be a provocative (uncommon) factor in the occurrence of TEN [6].

Intracranial malignancy is a condition that can be complicated by seizure activity. Antiepileptic drugs (AEDs) are typically employed as a prophylactic anticonvulsant agent; phenytoin and levetiracetam are the most common AED used for patients with intracranial malignancies and it is not uncommon for those patients to receive those drugs in conjunction with cranial radiation therapy. Toxic epidermal necrolysis (TEN) can develop in such patients during or soon after cranial radiation and can rapidly progress to erythema multiforme (EM) major [7].

## 2. Case Presentation

A 42-year-old Philipino male patient, with a history of hypertension, dyslipidemia, and unknown allergy, was diagnosed on August 2012 as a case of glioblastoma multiforme, after he presented with headache and confusion. The patient underwent tumour debulking surgery on August 25, 2012. He was started on oral phenytoin 300 mg once daily for prevention of epileptic attacks on August 28, 2012. phenytoin was started in hospital for 9 days before discharge and he continued on same dose at home. On October 1, 2012, the patient was started on (concomitant chemotherapy/radiotherapy) Temozolomide 75 mg/m^2^ po daily with radiotherapy (RT) daily (for 6 weeks); a regimen of dexamethasone (12 mg/day) and Trimethoprim/sulfamethoxazole DS was started for him, and on October 5 (after 5 days of chemotherapy/radiotherapy) he presented to urgent care with severe rash on the arms and shoulders; he stated that he started to have rash on his shoulders after 2 weeks on phenytoin but started to spread to his arms, back, chest, and both legs which became more generalized and associated with itching after he was started on radiotherapy. In urgent care he received antihistaminic and corticosteroids, phenytoin was discontinued and switched to levetiracetam 500 mg po twice daily, his rash improved, and he was discharged home. On October 7, he reported back to urgent care with erythema, pruritis, facial swelling, exfoliative dermatitis, and exanthematous rash on the face, arms, thighs, chest, and back with minimal mucosal involvement (Figure 1) along with fever and shivering, and then he was admitted to the hospital for treatment and observation; on admission his medications consisted of aspirin 100 mg oral tablet once daily for cardiovascular event prevention, Atorvastatin 20 mg oral tablet once daily for dyslipidemia, dexamethasone 4 mg three times daily as a part of his concomitant chemotherapy/radiotherapy protocol, Enalapril 5 mg oral tablet once daily for hypertension, Temozolomide 130 mg oral tablet once daily for glioblastoma, and Trimethoprim/sulfamethoxazole DS for pneumocystis jiroveci pneumonia (PCP) prophylaxis. After admission, we discontinued all potentially responsible drugs (levetiracetam) and radiotherapy and Temozolomide were held; he received diphenhydramine 50 mg intravenously STAT then once daily and corticosteroids were started (intravenous hydrocortisone 200 mg STAT, then maintained on dexamethasone 8 mg daily, and he was discharged on the same dose), even though their effectiveness has never been demonstrated in controlled trials for TEN treatment. During the hospitalization both dermal and general clinical conditions slowly improved, he was discharged home on October 15, and now he is followed as an outpatient in the clinic. 

## 3. Discussion

SJS and TEN are severe, acute, mucocutaneous reactions that are commonly induced by drugs. Drug-induced SJS and TEN typically begin 1 to 3 weeks after the initiation of therapy but occur more rapidly with drug rechallenge or in the presence of provoking factors. More than 100 different compounds have been implicated in both syndromes; barbiturates are some of the most common among them [7]. A case-control study was performed which clearly revealed that SJS and TEN are associated with short-term therapy with phenytoin, phenobarbital, and carbamazepine; in this study it was declared that the period of increased risk is confined to the first 8 weeks of treatment [8]. 

Risks for serious skin reactions vary considerably among AEDs [9]. A case-control study compared group of patients receiving phenytoin, carbamazepine, lamotrigine, and phenobarbital with control patients not receiving AEDs and resulted in relative risks of 50 to 130 for hypersensitivity syndrome, SJS, and TEN [10]. In 70% of patients, a cross-reaction of cytochrome P450-inducing aromatic amines (phenobarbital, phenytoin, carbamazepine, and lamotrigine) triggers hypersensitivity syndrome, SJS, and TEN.

Because of the variety and the rarity of adverse systemic reactions to AEDs, physicians initiating AEDs should counsel patients to notify their physician if they develop any new or unusual symptoms. The wide variety of adverse reactions of AEDs which highlights the number of body systems other than the CNS gives emphasis to the importance for physicians to evaluate all aspects of a patient's physical functioning during usage of AEDs.

## 4. Consent

Written consent was obtained from the patient for publication of this case report and any associated photograph. 

## 5. Conclusion

Although toxic epidermal necrolysis is a rare toxicity, it should always be considered with concurrent administration of phenytoin with cranial radiation therapy.

The need for prophylactic anticonvulsant therapy in patients undergoing cranial radiation therapy should be assessed on a case by case basis. If anticonvulsants are employed, then they must be administered with caution, and all cutaneous reactions developing subsequently within the radiation site must be promptly evaluated with a high index of suspicion for erythema multiforme. As the first suspicious sign is seen, antiepileptic drugs should be discontinued immediately and aggressive medical management should be started [7].

Because of the variety and the rarity of adverse systemic reactions to antiepileptic drugs (AEDs), physicians initiating antiepileptic drugs (AEDs) should counsel patients on the importance of notifying their physician if they develop any new or unusual symptoms.

## Figures and Tables

**Figure 1:**